# Preliminary research on flow rate and free surface of the accelerator driven subcritical system gravity-driven dense granular-flow target

**DOI:** 10.1371/journal.pone.0187435

**Published:** 2017-11-02

**Authors:** Xiaodong Li, Jiangfeng Wan, Sheng Zhang, Ping Lin, Yanshi Zhang, Guanghui Yang, Mengke Wang, Wenshan Duan, Jian’an Sun, Lei Yang

**Affiliations:** 1 College of Physics and Electronics Engineering and Joint Laboratory of Atomic and Molecular Physics of NWNU, Lanzhou, China; 2 Institute of Modern Physics, Chinese Academy of Sciences, Lanzhou, China; 3 School of Physical Sciences, University of Chinese Academy of Sciences, Beijing, China; University of Liverpool, UNITED KINGDOM

## Abstract

A spallation target is one of the three core parts of the accelerator driven subcritical system (ADS), which has already been investigated for decades. Recently, a gravity-driven Dense Granular-flow Target (DGT) is proposed, which consists of a cylindrical hopper and an internal coaxial cylindrical beam pipe. The research on the flow rate and free surface are important for the design of the target whether in Heavy Liquid Metal (HLM) targets or the DGT. In this paper, the relations of flow rate and the geometry of the DGT are investigated. Simulations based on the discrete element method (DEM) implementing on Graphics Processing Units (GPUs) and experiments are both performed. It is found that the existence of an internal pipe doesn’t influence the flow rate when the distance from the bottom of the pipe to orifice is large enough even in a larger system. Meanwhile, snapshots of the free surface formed just below the beam pipe are given. It is observed that the free surface is stable over time. The entire research is meaningful for the design of DGT.

## Introduction

**Fig 1:**
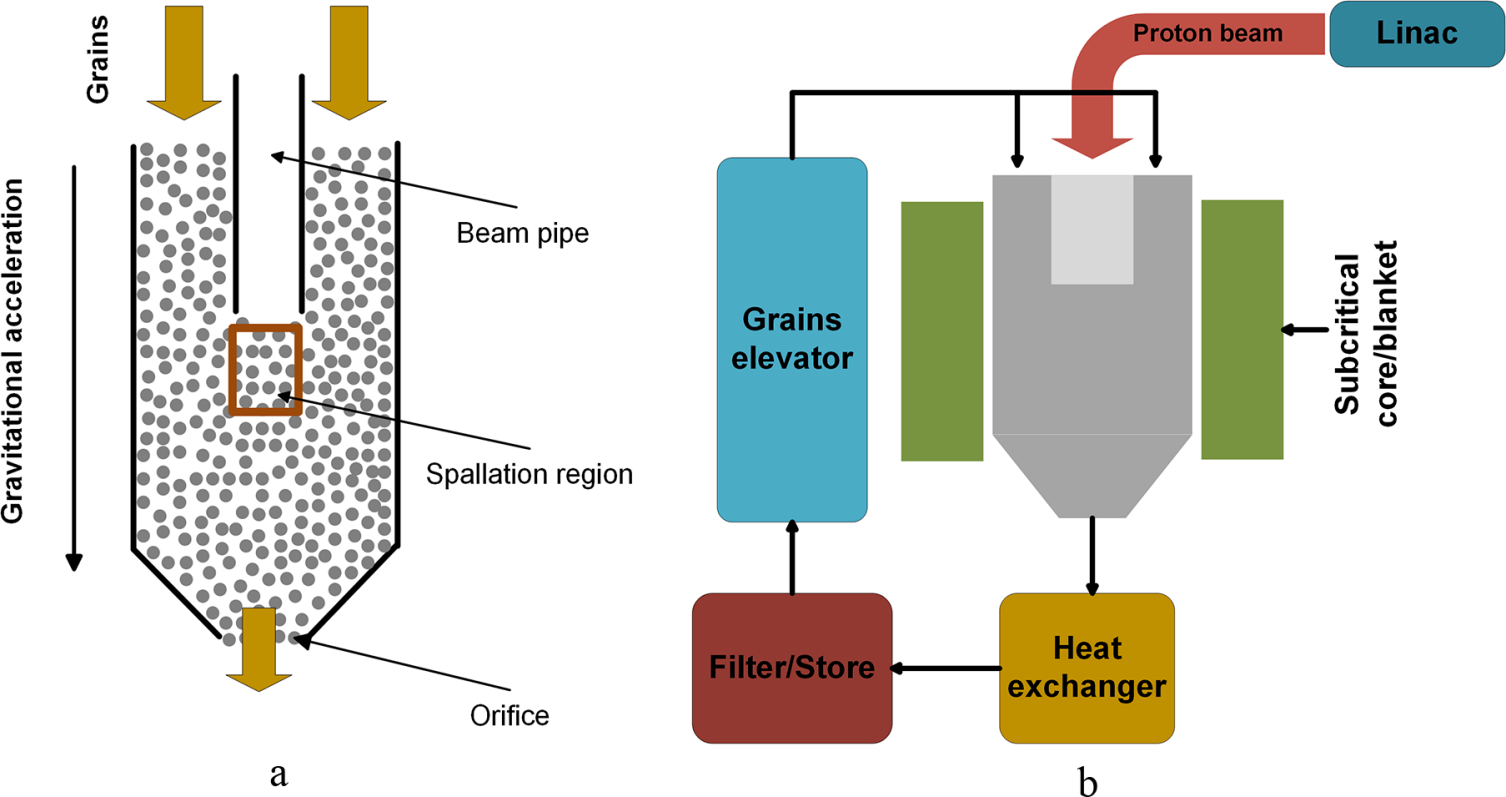
a: The simplified DGT; b: The sketch of ADS.

The accelerator driven subcritical system (ADS) (Fig 1B) consists of mainly three parts: an accelerator, which can provide high-intensity and high-energy proton beam; a spallation target, which can produce neutrons during the bombarding of the proton beam; a subcritical blanket, using the neutrons from spallation target to maintain nuclear reaction and transmuting minor actinides. Great efforts have been devoted to the R&D of the complex system in the last 30 years [1–4]. As reported by Riemer et al., the spallation targets can be divided into three categories: I, static solid targets, which are used in early designs in KENS, IPNS, WNR, ISIS; II, liquid targets such as in the Spallation Neutron Source (SNS), the Swiss Spallation Neutron Source (SINQ) in Paul Scherrer Institute (PSI) and in Japan Proton Accelerator Research Complex (J-PARC); III, the moving solid targets, including the wheel concept in Chinese Spallation Neutron Source (CSNS), the European Spallation Source (ESS) and the newly proposed granular flow concept in China Initiative Accelerator Driven System (CIADS) [5–7]. Among these targets, the gravity-driven Dense Granular flow Target (DGT) using tungsten grains is attractive in the aspects of high heat removal capacity, long operation life, as well as low chemical and radioactive toxicity [6].

In the hopper system (Fig 1A) of DGT, studying the flow rate is of great importance since flow rate is the key issue when discussing the heat removal and the wear of the grains. When the flow rate is large, the wear of the grains in the hoister will be considerable large due to different kinds of collisions; when the flow rate is small, the heat transfer of the system is limited since the velocity of the grains is small.

The proposed target consists of a cylindrical hopper and an internal coaxial cylindrical beam pipe (as shown in Fig 1A), which is more complicated than an ordinary hopper. The flow rate of grains discharging from ordinary hoppers has already been investigated by both experiments and simulations for a long time [8–12]. It is generally agreed that the flow rate is independent of the packing height provided *H* ≥ 2*D*, where *H* is the packing height and *D* is the diameter of the hopper. Furthermore, the flow rate is independent of the hopper diameter if *D* > 2.5*D*_0_ (*D*_*0*_ is the diameter of hopper orifice) [13]. Now, a widely accepted conclusion about the flow rate is that the flow rate can be described by the law proposed by Beverloo et al., which has the form $\varphi =C\rho_{b}\sqrt{g}{\left(D_{0}-kd\right)}^{2.5}$ for three-dimensional (3D) hoppers with round outlet and $\varphi =C\rho_{b}\sqrt{g}{\left(D_{0}-kd\right)}^{1.5}$ for two-dimensional (2D) hoppers. Here *C* and *k* are dimensionless coefficients, *g* is the acceleration of gravity, *ρ*_*b*_ is the equivalent density, and *d* is the diameter of the grain, respectively [9]. This law is valid whatever the hopper is conical or flat-bottomed. Here, for simplification, a flat-bottomed hopper with a round orifice is simulated [14, 15]. Although there are numerous studies about hopper flows, the details of flows in a hopper with an internal pipe is still unclear. Our previous numerical research showed that the beam pipe has no effect on the flow rate under certain conditions in a small-scale system [16]. However, the laws may be different in a granular system with different size due to the boundary effect. In this paper, simulations as well as experiments in large-scale systems have been performed to further study this issue.

A free surface could be formed below the internal pipe and the stability of the surface is another issue of great concern. Thermal hydraulic studies of the free surface about HLM targets have become important recently in the innovation nuclear reactors designs. The free surface formed in HLM targets has been researched by both simulations and experiments by some groups focusing on the target designs [17–21]. The multi-propose hybrid research reactor for high-tech applications (MYRRHA) is currently in design at Mol/Beigium. The MYRRHA’s first design was tested using water and the experiments were performed at the University Catholic de Louvain (UCL) [22]. Computational fluid dynamics (CFD) simulations, accounting for mass transfer across the free surface, were conducted with the Volume of Fluid method (VOF) [17, 23–27]. The experimental results from UCL demonstrated the capabilities of the VOF method. There are also other free-surface simulation methods, such as Arbitrary-Lagrangian-Eulerian Moving-Mesh Algorithm (ALE-MMA) [25] and the Large Eddy and Interface Simulations (LEIS). Furthermore, the Smoothed-Particle Hydrodynamics (SPH) approach employed as one of the methods for the spallation target design proposed by the European Spallation Source (ESS) is reported [17, 28]. Measurements showed that the surface in the target is stable in a wide range of operating conditions, apart from small surface waves and no major fluctuations of the free surface shape were observed [22]. Meanwhile, numerical results from the pre-calculations using CFD code Star-CD showed very good agreement with the experimental results of the observed free surface (the discrepancy is less than 5%) [22]. In the DGT, the research on the free surface is also of great importance. In this paper, DEM is used to simulate the flowing process of the grains in the target and to quantitatively describe the surface shape. Experimental techniques are also used to observe the free surface in the target.

## Methodology

### DGT and its simplified model & simulation method

The DGT and its simplified model can be found in the previous work [16] and the DEM is also introduced [29] in detail. The validity of the method has been checked in last publications [30–32]. To study the DGT, a DEM code performed on GPU was developed by our group, in which Hertz model is adopted to simulate the contact of two grains. The Hertz model is widely accepted in the simulation of particles so far. With this code, free fall arch, which mainly deals with the power law of flow rate in an hopper, as well as the influence of wall friction to the flow rate are investigated. About the DGT model, the results from small scale simulations are: if the distance (*H* in this paper) from the orifice of the hopper to the beam pipe is large enough (at least 2 times of the diameter of the hopper), the beam pipe did not influence the flow rate of the hopper; Particle-wall and particle-particle frictions do affect the flow rate but density and restitution coefficient of particles do not; The influence of Young’s modulus on flow rate can be neglected unless very soft particles are considered.

**Fig 2:**
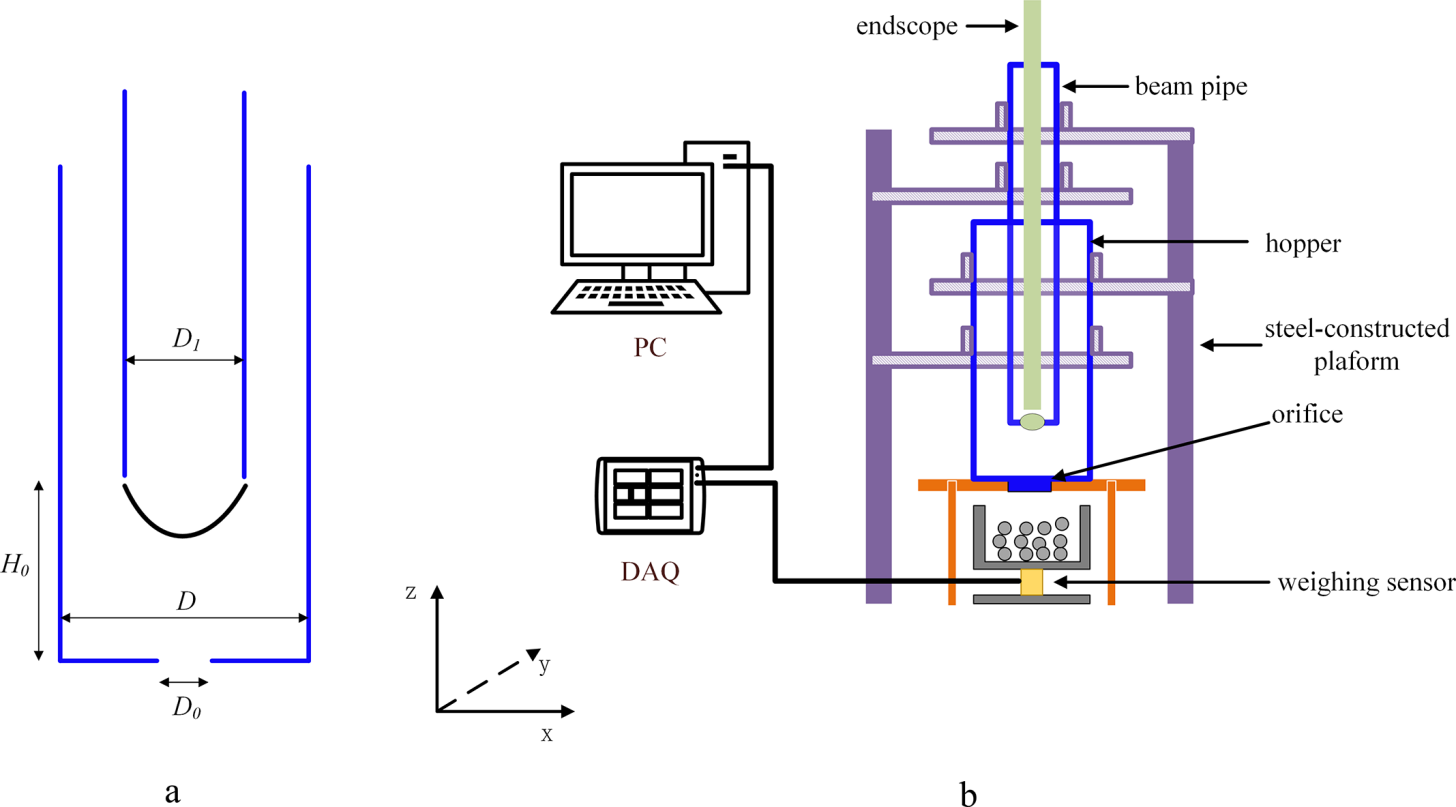
a: The sketch of DGT; b: Experimental setup.

In this paper, we adopt the flat-bottomed hopper (Fig 2A) in the study. The parameters in the simulations are shown in Table 1. In the simulations, 4 000 000 mono-sized spherical grains are randomly inserted into the unoccupied spaces within the hopper and out of the inner pipe initially. After that, all the grains fall freely and the packing process does not stop until the kinetic energy of the whole system is small enough. Finally, we open the orifice and let the grains flow out of the hopper. It should be mentioned that both in the simulations and experiments, no new grains are added to the hopper. In each simulation case, the simulation time is long enough to allow the flow to become stable. The flow rate is calculated from the slope of discharge profiles in the same time window.

**Table 1:** Parameters for the simulations and experiments.

Quantity	Symbol	Value
Young’s modulus (Gpa)	*E*	206
Coefficient of restitution	*ε*	0.95
Poisson’s ratio	*υ*	0.30
Grain-grain friction coefficient	*μ*_*pp*_	0.75
Grain-wall friction coefficient	*μ*_*pw*_	0.5
Grains density (kg/m^3^)	*ρ*	7800
Diameter of grains (m)	*d*	0.001
Distance from bottom of pipe to hopper’s orifice (*d*)	*H*	20,50,100,150,200
Diameter of hopper (*d*)	*D*	50,60,70, 90,100
Diameter of pipe (*d*)	*D*_*1*_	30,40,50,60,70
Orifice of hopper (*d*)	*D*_*0*_	8,16,24,32,40

Note: the young’s modulus, coefficient of restitution, poisson’s ratio, grain-grain friction coefficients and grain-wall friction coefficients are used in the simulations and in the experiments. These parameters can’t be determined accurately yet.

### Experimental setup

The whole experimental setup is shown in Fig 2B and it consists of four parts: 1, the circular tubes, made of Plexiglas, simulating the hopper and beam pipe in the real system. The size of these components are shown in Table 1 in the unit of the grains’ diameter. 2, the steel-constructed platform, which is used to adjust the positions of hopper and beam pipe accurately. 3, the endoscope which is inserted into the beam pipe to record the free surface in the area where the beam is supposed to firstly couple with the granular flow. 4, the weighing system, including the weighing sensor, data acquisition card, computer, etc.

In the X-Z panel, the purple rectangles denote the steel-constructed platform and the blue rectangular frames represent the hopper and beam pipe. The hopper and beam pipe are fixed to the platform and can be adjusted in X-Y panel in a certain scale with a precision of 0.02 mm, which ensures the coaxiality of them. Below the hopper there is a yellow frame where a replaceable hopper orifice is placed in. The Fe grains will flow down from the hopper and then go into the vessel under which there is the weighing sensor. The data of mass variation over time is acquired by Data Acquisition system and is sent to the computer. The flow rate is calculated from the slope of discharge profiles in the same time window. For the flow rate calculation, experiments have been performed for five times with the same settings for every certain geometry and the averaged value is taken as the final flow rate. Images of the free surface are recorded with endoscope.

## Results and discussions

### Relations of the flow rate with geometrical parameters

Here the influence of the internal pipe on the flow rate is investigated. In a hopper granular flow, jamming always happens when the orifice is less than 6*d* and the flow stops as a result [33]. Here the orifice is no less than 8*d* to avoid the jamming problem. To compare with the experiments, the mass flow rate, which is the mass of the grains pass through the orifice per second, is adopted in this study. In simulations and experiments, the initial packing height is at least 400*d* and not the same in all cases.

**Fig 3:**
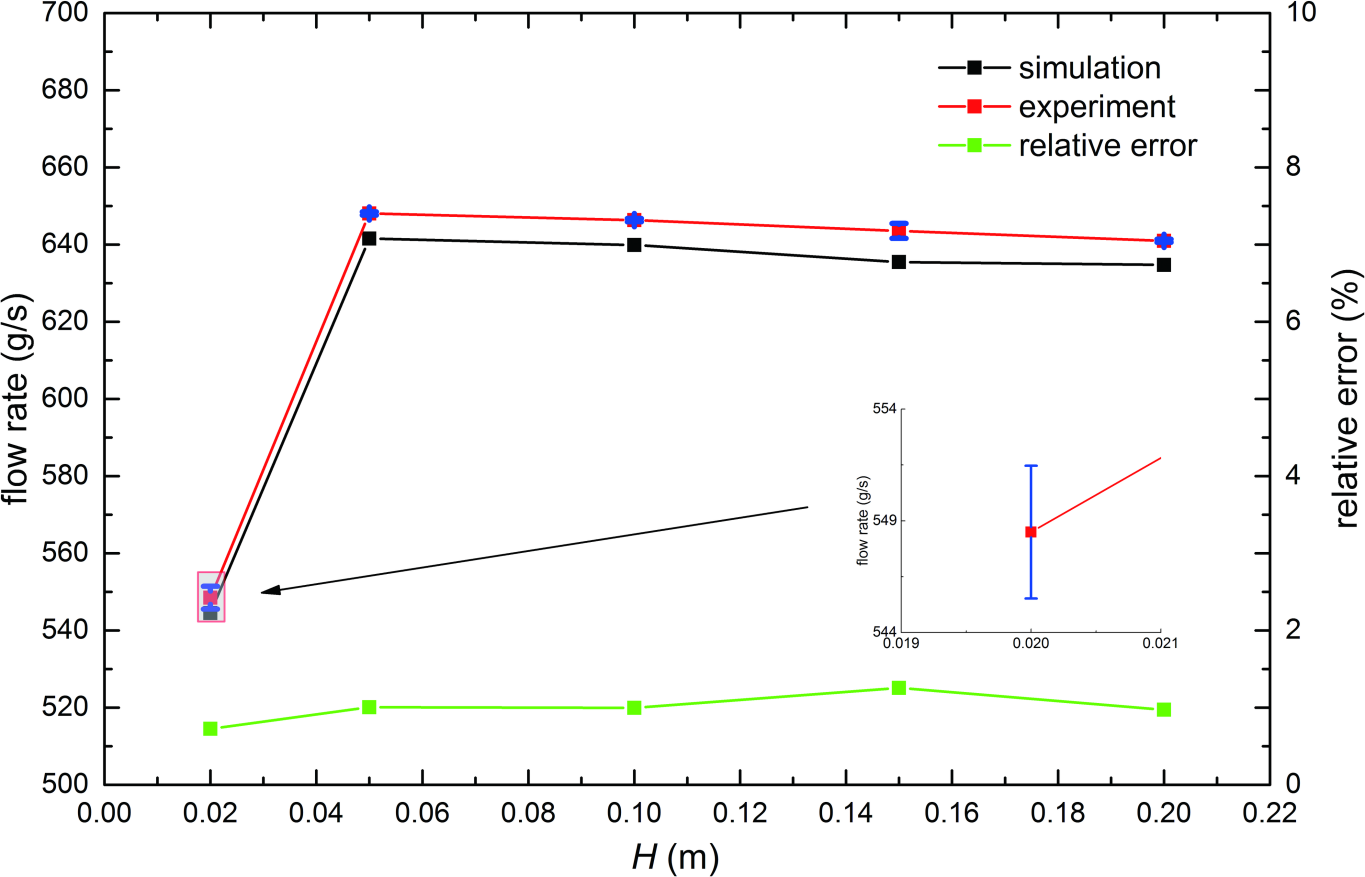
Relation of the flow rate with *H*. In the simulations and experiments. *H* ranges from 20 to 200*d* as Table 1 shows, while *D*, *D*_*0*_ and *D*_*1*_ are fixed to 100*d*, 24*d* and 50*d*, respectively. Error bars of experimental results are given in blue color and the largest error bar is magnified.

As is shown in Fig 3, the influence of the beam pipe is obvious when *H* is no less than 20*d* (20*d*, flow rate about 544 g/s), and is negligible when *H* exceeds 50*d* (flow rate about 640 g/s). As is reported by previous works, there are some regions in the hopper flows where the flow types are different and the mechanical structures near the orifice is the most important [11]. Therefore, it’s natural that when *H* is small the beam pipe can be regarded as an obstacle to hopper’s orifice and the influence can be neglected until *H* exceeds 50*d*. The results from the experiments have the same trends as that of simulation results and their difference is no more than 1.3%, which can be seen in Fig 3.

**Fig 4:**
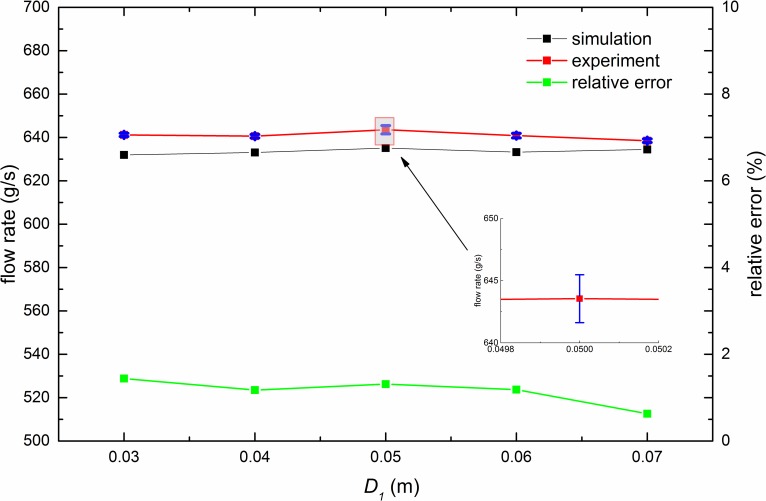
Relation of the flow rate with *D*_*1*_. In the simulations (experiments), *D*_*1*_ ranges from 30 to 70*d* (80*d*), while *D*, *D*_*0*_ and H are fixed to 100*d*, 24*d* and 200*d*, respectively. Error bars of experimental results are given in blue color and the largest error bar is magnified.

Fig 4 shows the relations of *D*_*1*_ with flow rate. It demonstrates that when *H* is large enough, i.e. *H* = 200*d*, variations of the pipe diameter do not bring change to the flow rate even when *D*_*1*_ = 70*d* and *D*-*D*_*1*_ = 30*d*. The difference between experiment and simulation is no more than 1.45%.

**Fig 5:**
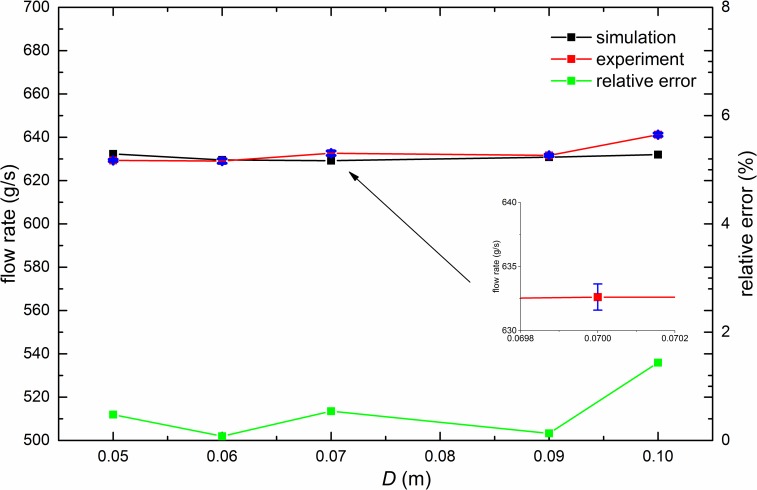
Relation of the flow rate with *D*. In the simulations (experiments), *D* ranges from 50*d* (40*d*) to 100*d*, while *D*_*1*_, *D*_*0*_ and *H* are fixed to 30*d*, 24*d* and 200*d*, respectively. Error bars of experimental results are given in blue color and the largest error bar is magnified.

The dependence of flow rate on hopper diameter *D* is presented in Fig 5. The results indicate that *D* does not influence the flow rate in such a configuration. The difference between experiment and simulation is no more than 2.0%.

**Fig 6:**
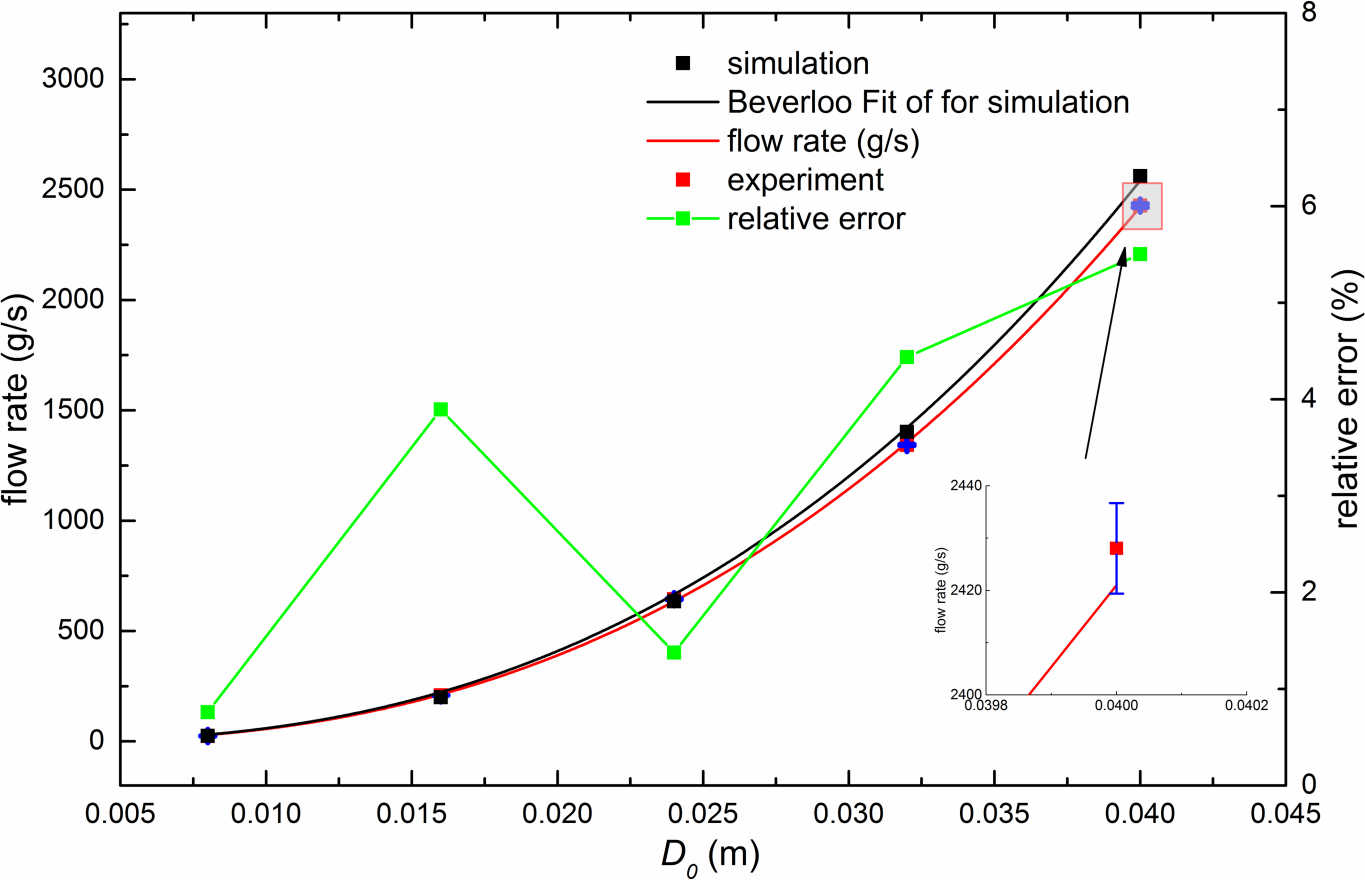
Relation of the flow rate with *D*_*0*_. In the simulations (experiments), *D*_*0*_ ranges from 8*d* to 40*d*, while *D*_*1*_, *D* and *H* are fixed to 50*d*, 100*d* and 200*d*, respectively. Error bars of experimental results are given in blue color and the largest error bar is magnified.

Fig 6 shows the fitting of the flow rate with *D*_*0*_ for the system. In the fitting with Beverloo’s law, it can be seen that the fitting is quite good. For the experimental results, fitted parameter *C* is 0.565 and *k* is 1.42, while for simulation results, they are 0.593 and 1.42, respectively. These parameters, with such values, are consistent with previous studies [11, 34].

The trends in Figs 3–6 are consistent with those shown in the previous research, even though the size of the geometry in this paper is much larger [16].

### Configuration of the free surface

This section will describe the free surfaces’ shape with a fitted formula first and then give their fluctuation calculations. Velocity field will be shown and discussed.

**Fig 7:**
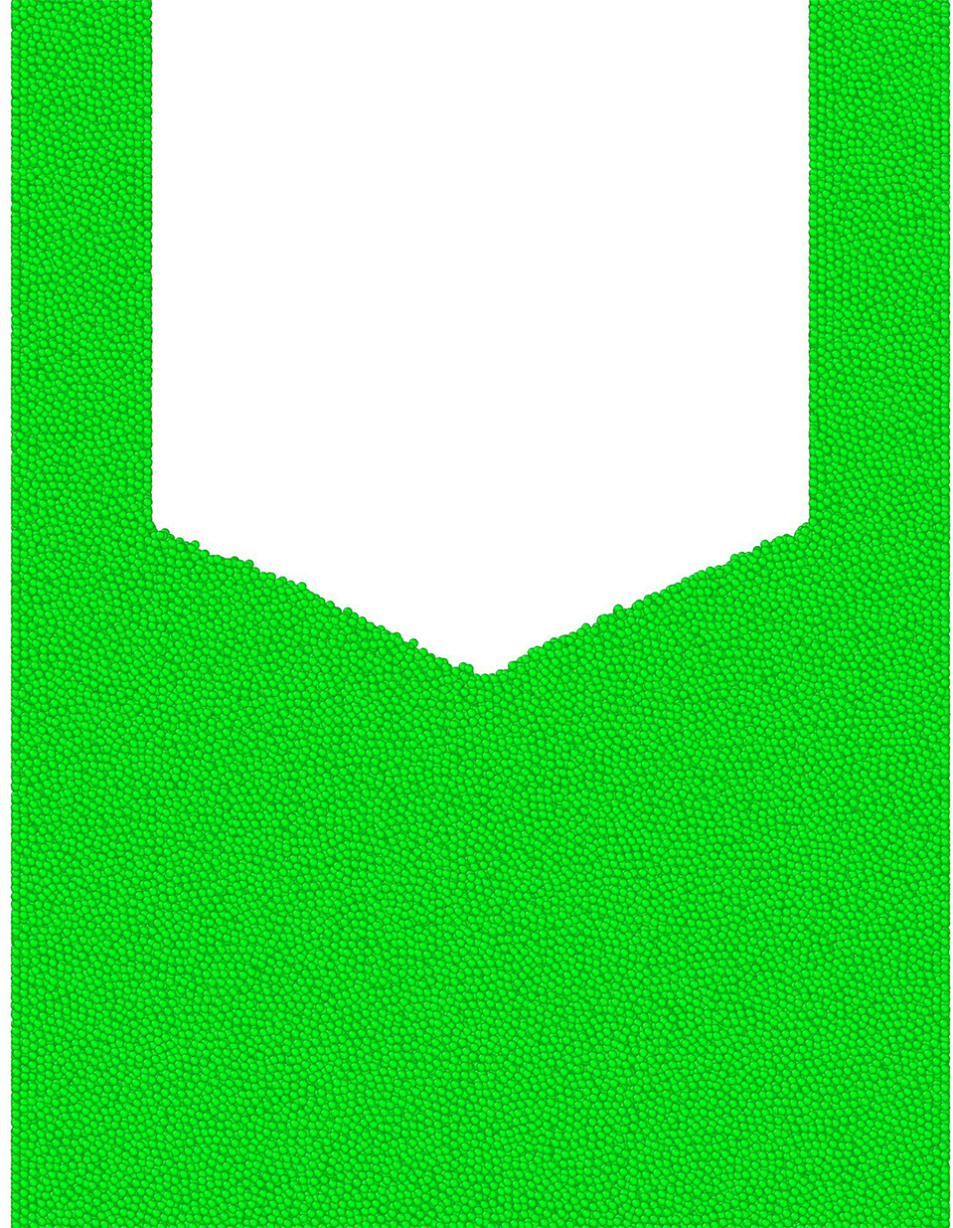
Sketch map of the free surface. A slice with the width 0.005 m in the flowing grain system.

**Fig 8:**
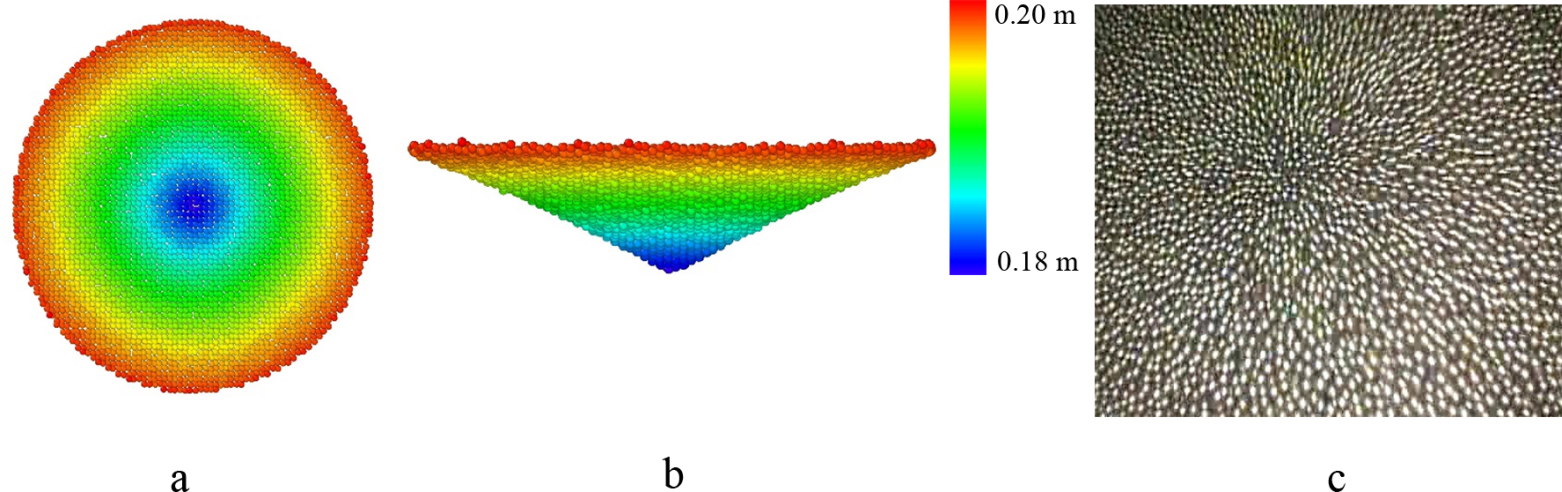
**Free surface in simulation and experiment:** a, top view of averaged free surface from simulation; b, front view of averaged free surface from simulation; the color in the map represent the height in Fig a & Fig b; c, top view of one snapshot free surface from experiment. For these figures, parameters are *H* = 200*d*, *D* = 100*d*; *D*_*1*_ = 70*d*; *D*_*0*_ = 24*d*, respectively.

**Fig 9:**
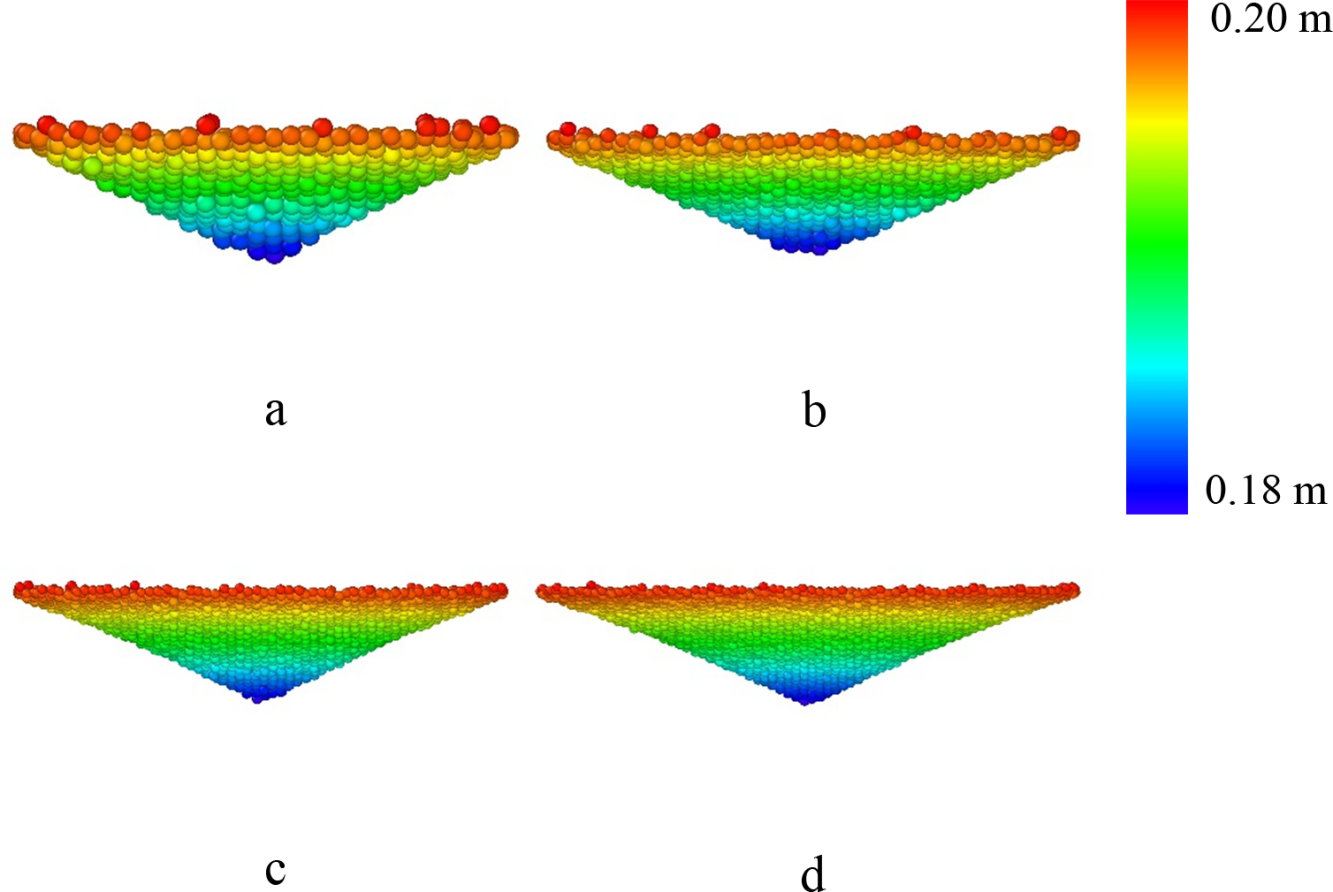
**Front views of averaged free surface with different pipe diameter:** a, *D*_*1*_ = 30*d*; b, *D*_*1*_ = 40*d*; c, *D*_*1*_ = 60*d*, d, *D*_*1*_ = 70*d*. The other parameters are *H* = 200*d*, *D* = 100*d*, *D*_*0*_ = 24*d*, respectively.

To plot the free surface, data of ten contiguous snapshots in the flowing process are extracted from the simulations. The time gap of the snapshots is 0.1 second. Fig 7 shows the simulation results of one snapshot and the unconfined grains under the beam pipe form a rough shape, i.e. the free surface. For the ten simulation snapshots, free surface is calculated for each of them. This method is quite like the one used to describe the external contour of converging flow and recirculation zone in the water experiment in the HLM targets [20]. The averaged free surface is shown in Fig 8(A) and 8(B) with different visual angles. Fig 8C shows the grains on the free surface in the experiment. Some of the grains in the endoscope are not spheres due to the big velocity of them. In Fig 9, it can be observed that the shape with different beam pipe’s diameters are nearly the same, which is like a surface by rotating a parabola around z axis. The shapes are fitted with the formula given as below:

$$z=A{\left(\sqrt{x^{2}+y^{2}}-C\right)}^{2}+B$$ (1)

**Fig 10:**
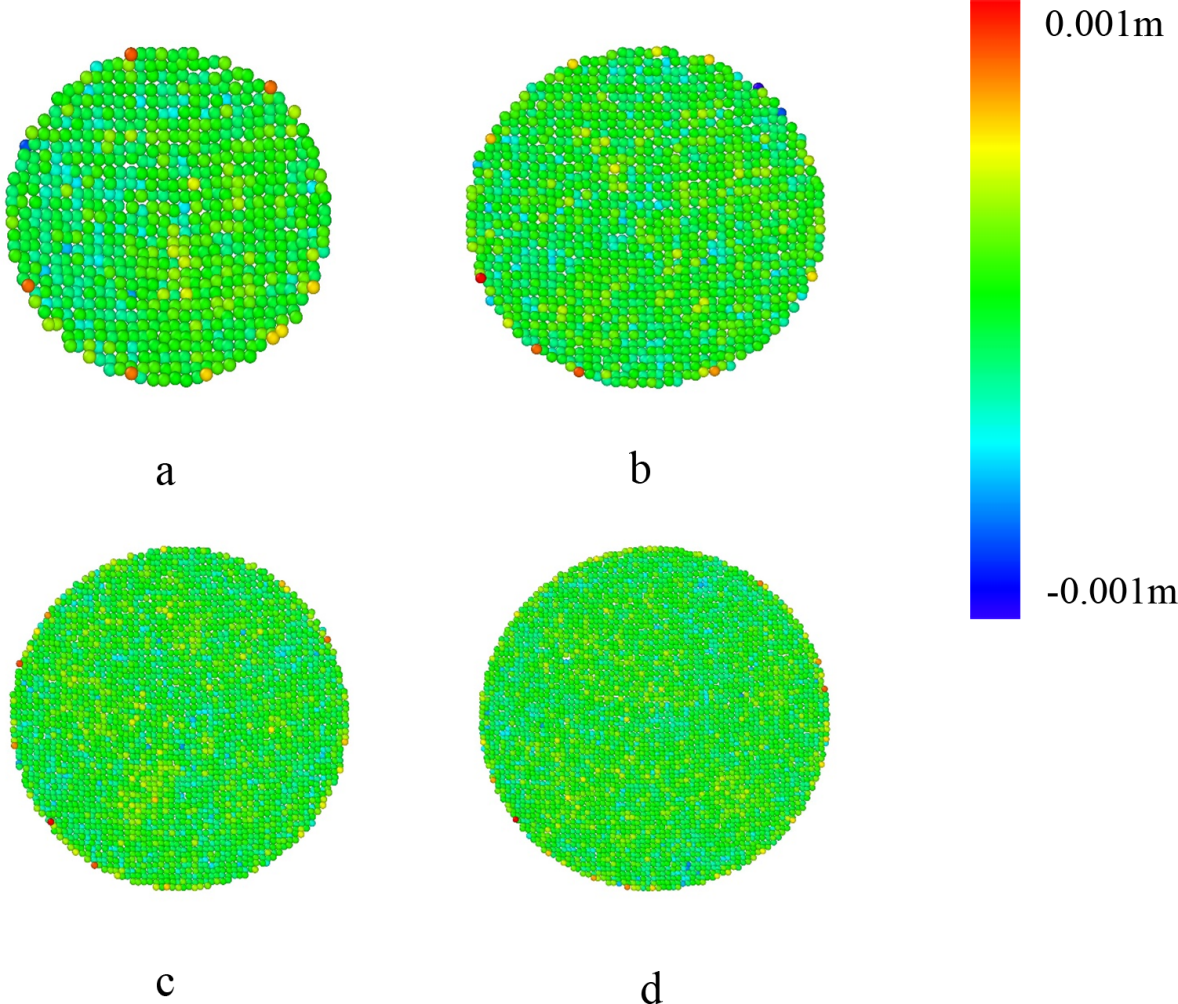
**δz of the fitted free surface and the grains with different pipe diameter:** a, *D*_*1*_ = 30*d*; b, *D*_*1*_ = 40d; c, *D*_*1*_ = 60d, d, *D*_*1*_ = 70*d*. The other parameters are *H* = 200*d*, *D* = 100*d*, *D*_*0*_ = 24*d*, respectively.

Fig 10 shows the discrepancy between the fitted surfaces from simulations with the averaged grains’ coordinate *z*_0_. The parameters *A*, *B* and *C* are calculated in the fitting procedure, then, *z*(*x*, *y*) is obtained. The *δz* is calculated by *z*_0_-*z*(*x*, *y*). From Fig 10, it’s obvious that almost all *δz* are less than 0.001m (the diameter of the grains). Table 2 shows the parameters in the fitting for different pipe’s diameters. The parameters are different for different pipes, but the values are fluctuating within a small range. It can be concluded that the formula is quite suitable for these free surfaces in this condition.

**Table 2:** Fitted parameters with formula 3.

D_1_	30	40	50	60	70
A	-1.66155	-1.71523	-2.20836	-2.88755	-2.99897
B	0.23461	0.22718	0.21742	0.21047	0.20952
C	0.16253	0.14937	0.11752	0.09449	0.09581

Note: Table 2 shows the fitted parameters in the formula (3) for the free surfaces. In the table, D1 changes from 30d to 70d while other parameters are H = 200d, D = 100d, D0 = 24d, respectively.

**Fig 11:**
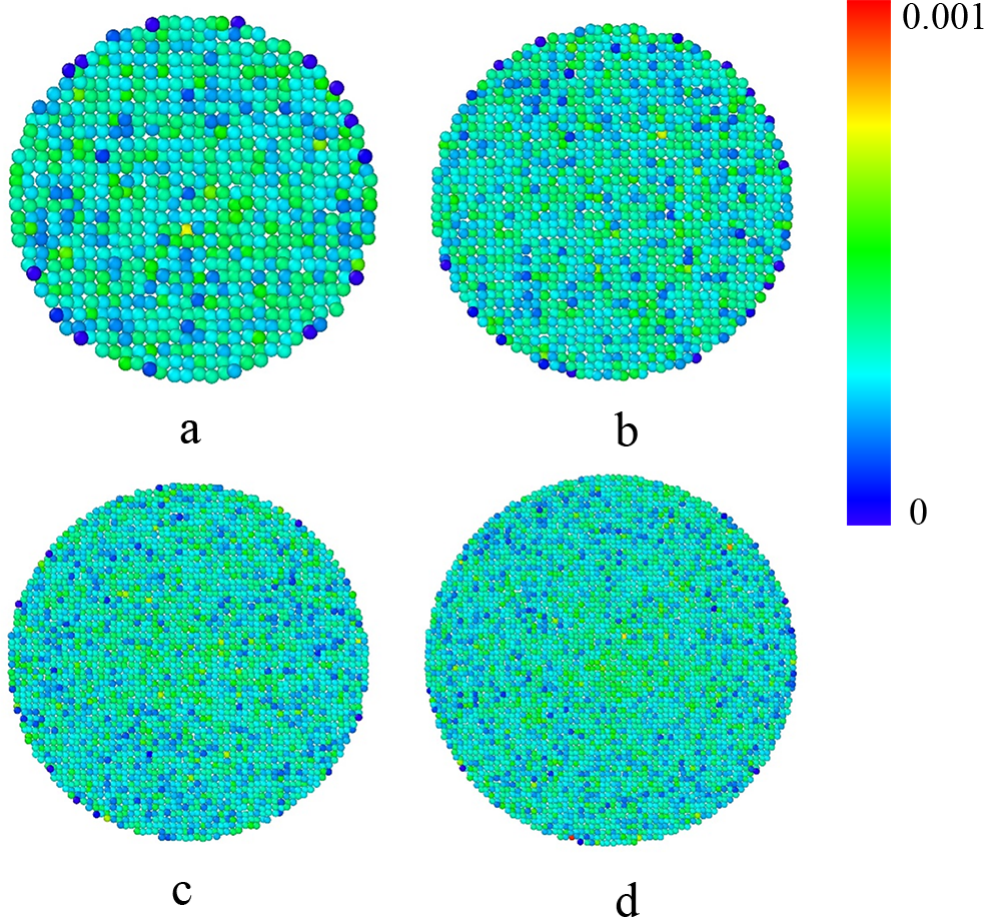
**Standard deviation of the free surface with different pipe’s diameter:** a, *D*_*1*_ = 30*d*; b, *D*_*1*_ = 40*d*; c, *D*_*1*_ = 60*d*, d, *D*_*1*_ = 70*d*. The other parameters are *H* = 200*d*, *D* = 100*d*, *D*_*0*_ = 24*d*, respectively.

Fig 11 shows the fluctuation of the ten free surface calculated above. It can be seen that the standard deviation of the z coordinates is just in the order of 10^−3^. We can conclude that most of the grains on free surfaces are fluctuating within a small range, at about the level of *d* (*d* = 10^−3^ m). The results reveal that the free surface can be stable as time goes on in the configuration where *H* is large enough (at least two times of the diameter of the hopper).

**Fig 12:**
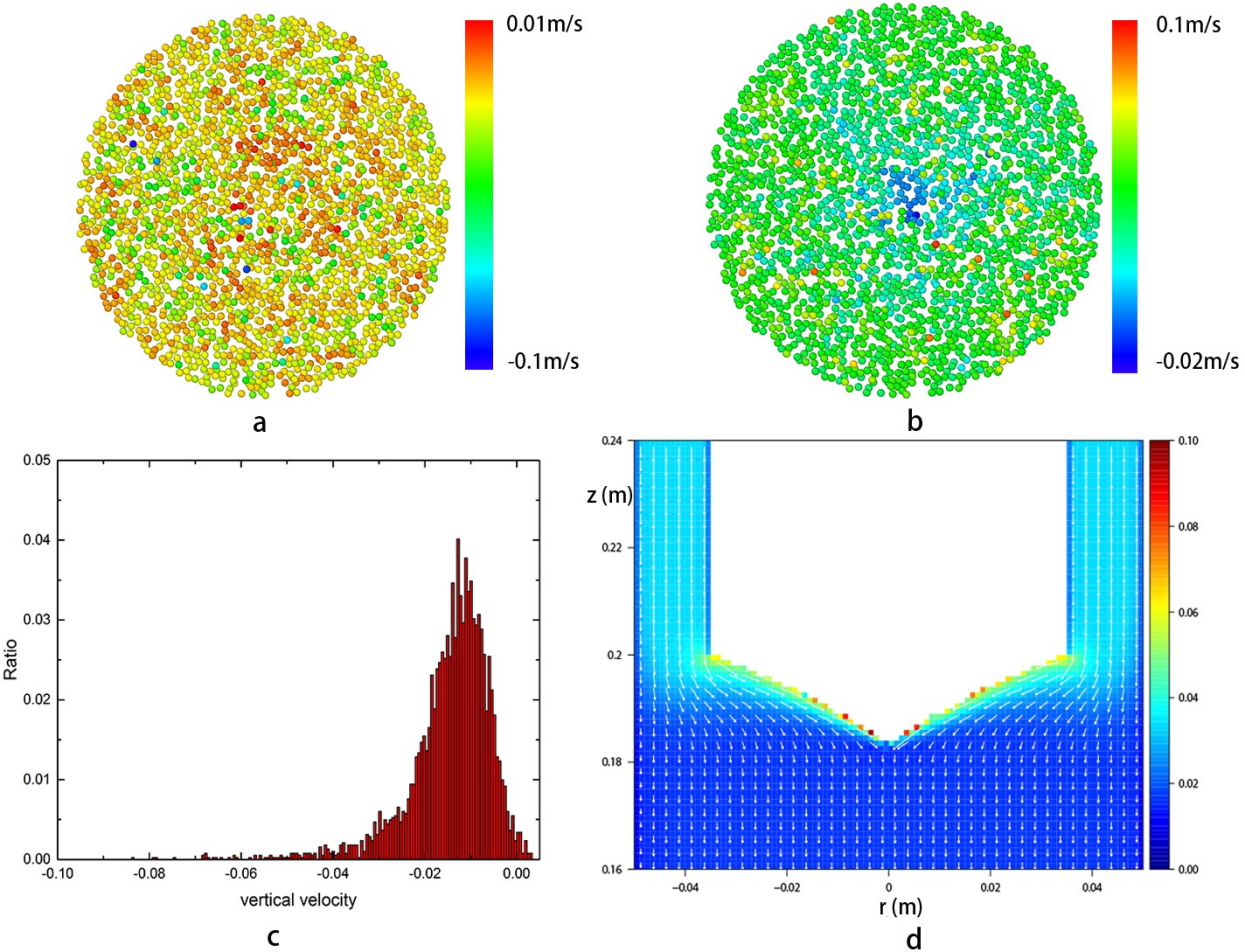
**Velocity field of grains on the free surface:** a, vertical velocity filed; b, radical velocity field. c, the distribution of the vertical velocity. d, resultant velocity calculated from vertical velocity and radical velocity. In this Fig, Both radical and vertical directions are the same as that in the cylindrical coordinate system. The parameters are *H* = 200*d*, *D* = 100*d*, *D*_*1*_ = 70*d*, *D*_*0*_ = 24*d*, respectively.

The simulation results show that most of the grains on the free surface gather to the center of the surface while flowing down. When getting to the center, these grains drop quickly and are covered by the coming grains on the surface immediately. The vertical and radical velocity field of the grains on the free surface are shown in Fig 12A and Fig 12B. It shows that there are grains whose vertical velocities are above zero, which means that some grains could move upward. Fig 12B tells that the radical velocity of the grains on free surface ranges from -0.02 m to 0.1 m/s, among which most are at about 0.05 m/s. Fig 12(C) gives the quantitative distribution of grains vertical velocity. It can be observed that percentage for the grains with upward velocity is less than 1.2% and their values are below 0.001 m/s in this case. Fig 12D shows the resultant velocity of the grains, which is calculated from Fig 12A and 12B. It can be observed that some grains on the free surface has the largest velocity and no recirculation area exists in this field. This is quite different from the results obtained in the HLM targets.

In the spallation target, the beam will interact with the grains on the surface first and deposit large quantity heat on them simultaneously. The power of beam deposition is stable over time, so the duration of stay of the grains on the free surface (or in the penetration depth of the proton beam) is a key factor which determines the highest temperature of the grains in the system. As a result, the grains with upward velocity would be heated to high temperature when the target is running. From this perspective, the optimization of the geometry, aiming to eliminate these kind of grains should be done in the design of the target.

## Conclusions

This work studies the flow rate and free surface of the DGT with DEM simulations and experiments. The results demonstrate that if *H* is large enough (at least two times of the diameter of the hopper), the influence of the beam pipe can be neglected and the flow rate of the DGT can be regarded as the same with an ordinary hopper even in larger system. It’s found that under the conditions above, the Beverloo’s law is still valid to predict the flow rate. Simulation results are consistent with that from the experiments. It can be concluded that increasing the size of the hopper doesn’t change the effectiveness of the laws about flow rate in this system.

The free surface formed in the supposed beam coupling zone is analyzed and the corresponding velocity field is extracted. The fluctuation of the free surface is no more than 1*d* and can be regarded as stable in this studied system. The results in this paper are meaningful to the configuration design of the DGT. Since the free surface is very important in the target design, more factors (such as the size of target, spallation materials, high temperature and proton beam coupling) should be considered. So far, a beam target coupling program has already been developed and much more work will be done in the future.

## Abbreviations

E: Young’s Modulus of grain’s material
ε: Coefficient of restitution
υ: Poisson’s ratio
μ_pp_: Grain-grain friction coefficient
μ_pw_: Grain-wall friction coefficient
ρ: Grains density
d: Diameter of grains
H: Distance from bottom of pipe to hopper’s orifice
D: Diameter of hopper
D_1_: Diameter of pipe
D_0_: Orifice of hopper
z: z coordinate of averaged free surface
z_0_: fitted z coordinate of averaged surface
δz: z_0_ -z
